# The impact of Er:YAG laser enamel conditioning on the microleakage of a new hydrophilic sealant—UltraSeal XT® hydro™

**DOI:** 10.1007/s10103-016-1878-y

**Published:** 2016-03-10

**Authors:** Z. A. Güçlü, N. Dönmez, T. Tüzüner, M. E. Odabaş, A. P. Hurt, N. J. Coleman

**Affiliations:** Diş Hekimliği Fakültesi Pedodonti, Erciyes Üniversitesi, ABD 38039 Melikgazi Kayseri, Turkey; Bezmialem Vakıf Üniversitesi, Diş Hastalıkları ve Tedavisi, ABD Adnan Menderes Bulvarı Vatan Caddesi, 34093 Fatih, İstanbul Turkey; Diş Hekimliği Fakültesi Pedodonti, Karadeniz Teknik Üniversitesi, ABD Kanuni Kampüsü, 61080 Trabzon, Turkey; Diş Hekimliği Fakültesi Dekanlık, Gazi Üniversitesi, Bişkek Caddesi 82 Sokak No:4 06510 Emek, Ankara, Turkey; Faculty of Science and Engineering, University of Greenwich, Chatham Maritime, Kent, ME4 4TB UK

**Keywords:** Er:YAG laser, Hydrophilic fissure sealant, Microleakage, Scanning electron microscopy

## Abstract

UltraSeal XT® hydro™ is a new hydrophilic, light-cured, methacrylate-based pit and fissure sealant which has been developed by Ultradent Products, USA. The sealant is highly filled with a 53 wt.% mixture of inorganic particles which confer both thixotropy and radiopacity. The principal purpose of this study was to investigate the microleakage of UltraSeal XT® hydro™ as a function of different enamel etching techniques. The occlusal surfaces of sound, extracted human molars were either acid etched, Er:YAG laser irradiated or successively laser irradiated and acid etched. UltraSeal XT® hydro™ was applied to each group of teeth (*n* = 10) which were subjected to a thermocycling process consisting of 2500 cycles between 5 and 50 °C with a dwell time of 30 s. Microleakage assessments were then carried out using 0.5 % fuchsin dye and optical microscopy. The microleakage score data were analysed using the Kruskal-Wallis, Mann–Whitney *U* test with Bonferroni adjustment. No significant differences in microleakage were noted between the individually acid etched and laser-irradiated groups (*p* > 0.05); however, teeth treated with a combination of laser irradiation and acid etching demonstrated significantly lower microleakage scores (*p* < 0.001). Electron microscopy with energy-dispersive X-ray analysis revealed that the mineral filler component of UltraSeal XT® hydro™ essentially comprises micrometre-sized particles of inorganic silicon-, aluminium- and barium-bearing phases. Laser etching increases the roughness of the enamel surface which causes a concentrated zoning of the filler particles at the enamel-sealant interface.

## Introduction

Due to their intricate morphology, occlusal pits and fissures are particularly vulnerable to caries and currently account for approximately 90 % of all caries-affected tooth surfaces [1]. Microorganisms, plaque and organic food debris which accumulate in pits and fissures are anatomically protected from a ready supply of remineralising saliva and are defended against routine brushing. For the past 30 years, a widely applied strategy in the prevention of pit and fissure caries has been to isolate these surfaces from the oral environment by the application of resin-based or glass-ionomer sealants [1–8].

The success of fissure sealants principally depends upon the quality of adhesion between the sealant and enamel which determines their ongoing resistance to the microleakage of saliva and microorganisms at the interface [1–8]. The adhesion and retention of the sealant are essentially derived from micromechanical interlocking, as very little chemical interaction exists between the resin and the enamel.

Tooth enamel is an avascular hard tissue comprising ∼96 % substituted hydroxyapatite (Ca_5_(PO_4_)_3_(OH)) nanocrystals arranged as rods (aka *prisms*) and ∼4 % water and trace proteins. The enamel surface is coated with a protective pellicle film of glycoproteins from saliva which can subsequently become colonised by oral bacteria and their breakdown products to form plaque. The formation of dental caries commences with the dissolution of hydroxyapatite under the action of organic acids produced by the fermentation of carbohydrates by cariogenic bacteria located in the plaque.

Prior to the successful application of a fissure sealant, the pellicle, plaque and organic debris are removed by brush and pumice paste. The enamel surface is then roughened to increase the surface area available for the mechanical interlocking of the sealant. Enhanced surface roughness can be achieved by direct mechanical abrasion with a metal bur, air abrasion with a sputtered jet of particles, acid etching or laser ablation [3–8].

The most widely accepted enamel conditioning procedure prior to the application of fissure sealants is exposure to 37 % phosphoric acid for 15 s, which selectively erodes the hydroxyapatite rods [3]. Practical disadvantages of phosphoric acid etching are that it is time consuming and requires the isolation of the tooth with cotton wool or a rubber dam [3, 9]. Remnants of debris and pellicle, the presence of aprismatic enamel and restricted entry into fissures can also reduce etching performance which compromises the quality of adhesion and longevity of the sealant [3, 4, 7].

Laser ablation has been nominated as an alternative enamel preparation technique for use with fissure sealants [4, 6, 7]. The absorption of laser energy by sub-surface water molecules within the enamel is accompanied by rapid heat evolution and volume expansion which causes micro-explosions on the enamel surface. One clinical advantage of laser ablation is that it is relatively rapid and does not require the isolation of the tooth [3, 7]. Laser etching is also reported to be more effective at conditioning aprismatic enamel and also more efficient in the removal of residual plaque and organic debris [4, 7]. Other potential benefits may include enhanced surface roughness and an increase in the calcium:phosphorus ratio of the lased hydroxyapatite which is associated with superior acid resistance and caries inhibition [4, 10].

A number of recent studies has been carried out to compare the etching efficiency of Nd:YAG, Er:YAG and Er, Cr:YSGG laser conditioning with that of conventional acid etching [3, 4, 6, 7, 9]. These reports present conflicting opinions on the impact of laser ablation on the retention and microleakage of resin-based fissure sealants. This lack of consensus appears to derive from the potential suitability of the technique as a function of the rheological and physicochemical properties of different sealants. It is suggested that, of the currently available sealants, less viscous, more ‘flowable’ systems may be better suited to the rougher surfaces presented by lased enamel.

UltraSeal XT® hydro™ is a new hydrophilic, light-cured, methacrylate-based pit and fissure sealant which has been developed by Ultradent Products, USA [11]. The sealant is highly filled with a 53 wt.% mixture of inorganic particles which confer both thixotropy and radiopacity. By virtue of its thixotropic nature and hydrophilic monomers, this material is reported to ‘chase’ moisture into the pits and fissures thus eliminating moisture-related failure which is associated with hydrophobic sealants [11].

The principal purpose of this study was to investigate the in vitro microleakage of UltraSeal XT® hydro™ as a function of different enamel etching techniques. The occlusal surfaces of sound, extracted human molars were either acid etched, Er:YAG laser ablated or successively lased and acid etched. UltraSeal XT® hydro™ was applied to each group of teeth (*n* = 10) which were subjected to a thermocycling process consisting of 2500 cycles between 5 and 50 °C. The teeth were then exposed to 0.5 % basic fuchsin dye solution for 24 h, sectioned into three slices in the bucco-lingual direction and examined for dye penetration (i.e. microleakage) using optical microscopy. The microleakage score data were analysed using the Kruskal-Wallis test and Mann–Whitney *U* test with Bonferroni adjustment. The sealed teeth were also investigated by scanning electron microscopy with energy-dispersive X-ray analysis.

## Materials and methods

Thirty sound, extracted human molar teeth were obtained from patients with orthodontic or periodontal problems who had tendered their informed consent. Ethical approval for this project was obtained on 1 October 2014 by the Ethical Committee of Bezmiâlem Vakif University (reference number 71306642/050-01-04/282) which was performed in accordance with the ethical standards laid down in the 1964 Declaration of Helsinki and its later amendments. The teeth were debrided with manual scaling instruments, cleaned with bristle brush and pumice paste and stored in distilled water for up to 4 weeks. The teeth were then randomly divided into three groups.

The occlusal surfaces of group I teeth were acid etched with 35 % phosphoric acid gel (UltraSeal XT® hydro™, Ultradent Products, USA) for 20 s, rinsed and lightly air dried, as suggested by the manufacturer. The UltraSeal XT® hydro™ sealant was then applied by the same operator, according to the manufacturer’s instructions, and light cured for 20 s (using a BA Optima 10 curing light, BA International Ltd., UK). The composition of the UltraSeal XT® hydro™ sealant, as listed in the safety data sheet provided by the manufacturer [12], is given in Table 1.

**Table 1:** Composition of UltraSeal XT® hydro™

Compound	Quantity (wt.%)
Triethylene glycol dimethacrylate	<20
Diurethane dimethacrylate	<8
Aluminium oxide	<4
Methacrylic acid	<1
Titanium dioxide	<0.3
Sodium monofluorophosphate	<0.2

Laser conditioning of the occlusal surfaces of group II teeth was carried out using an Er:YAG laser system (LightWalker®, Fotona, Slovenia) operating at a wavelength of 2940 nm, a power output of 1.2 W, pulse energy of 120 mJ and a frequency of 10 Hz. Laser ablation was carried out using a 600-μm-diameter sapphire tip with a beam spot size of 0.63 mm^−2^, energy density of 19 mJ cm^−2^ at a working distance of 8 mm at an angle of 90° under water cooling at 50 cm^3^ min^−1^. The lased teeth were rinsed with water and lightly air dried prior to sealing with UltraSeal XT® hydro™.

Group III teeth were sequentially subjected to laser ablation and acid etching, as described above, prior to the application and curing of the UltraSeal XT® hydro™ sealant.

Immediately after sealing, the teeth were placed in distilled water at 37 °C for 24 h and then thermocycled 2500 times between 5 and 55 °C with a transfer time of 10 s and a dwell time of 30 s [3, 6, 7]. Subsequent microleakage was assessed *via* the dye penetration method. The teeth were coated with nail varnish, leaving a 2-mm window around the sealant, and the roots were embedded in an acrylic resin cylinder (Meliodent, Bayer Dental, UK). The teeth were then placed in 0.5 % basic fuchsin dye solution for 24 h. Following immersion, the teeth were rinsed under running tap water for 5 min to remove excess dye and sectioned in the bucco-lingual direction using a water-cooled diamond saw to obtain three slices. Each of the tooth sample slices was then examined twice under a stereomicroscope (SMZ 800, Nikon, Japan) at 20× magnification by two investigators who were unaware of the pre-treatment of each sample. The microleakage scoring criteria [3, 4] are listed in Table 2.

**Table 2:** Microleakage scoring criteria

Score	Definition
0	No dye penetration
1	Dye penetration up to ½ of the fissure
2	Dye penetration beyond ½ of the fissure without total involvement
3	Dye penetration to the sealant base

The microleakage data were analysed using the statistical package SPSS 14.0.0 for Windows (SPSS, Chicago, USA). Inter-examiner reproducibility was analysed with the kappa statistic. Median differences among the microleakage data for each of the groups were compared using the Kruskal-Wallis test (*p* = 0.05). Significant differences were evaluated using the Mann–Whitney *U* test with Bonferroni adjustment (*p* = 0.05).

Scanning electron microscopy was carried out on the middle slices of the sectioned teeth using uncoated samples attached to carbon tabs on a JEOL JSM-5410 LV electron microscope with an Oxford Instruments X-MaxN EDX detector in low vacuum mode. All back-scattered electron images and EDX maps were obtained with an accelerating voltage of 20 kV at a working distance of 20 mm.

## Results

**Fig. 1:**
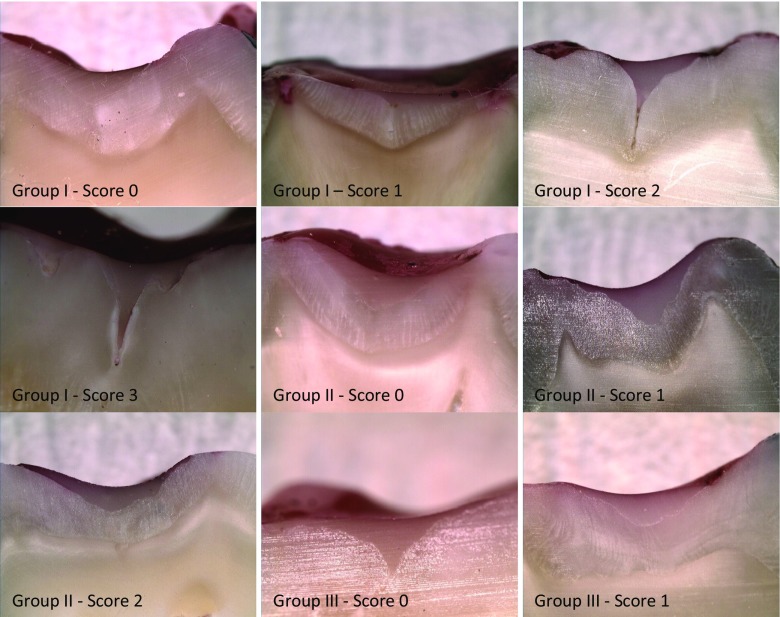
Images of sectioned teeth showing various levels of dye penetration

The distribution of microleakage scores for each experimental group is listed in Table 3, and images of the sectioned teeth showing various levels of dye penetration are given in Fig. 1. No significant difference in marginal leakage was found between the separately acid-etched (group I) and lased (group II) teeth (*p* > 0.05). In contrast, teeth conditioned by sequential laser irradiation and acid etching (group III) demonstrated significantly lower microleakage scores (*p* < 0.001) than the other two experimental groups. The maximum extent of dye penetration in group I teeth involved the complete fissure to the sealant base. In group II, maximum dye penetration extended beyond the mid-point but without total involvement; and only one sample in group III exhibited microleakage, with maximum dye ingress within the top half of the fissure. An inter-examiner kappa statistic of 0.92 was obtained for the microleakage evaluation which indicates high reproducibility.

**Table 3:** Distribution of microleakage scores as functions of etching regime

Etching regime	0	1	2	3	Significance^a^
Acid (group I)	17	3	5	5	a
Laser (group II)	15	13	2	0	a
Acid and laser (group III)	29	1	0	0	b

^a^Different letters indicate significant differences between groups (*p* < 0.001), and the same letter indicates no significant difference (*p* > 0.05)

**Fig. 2:**
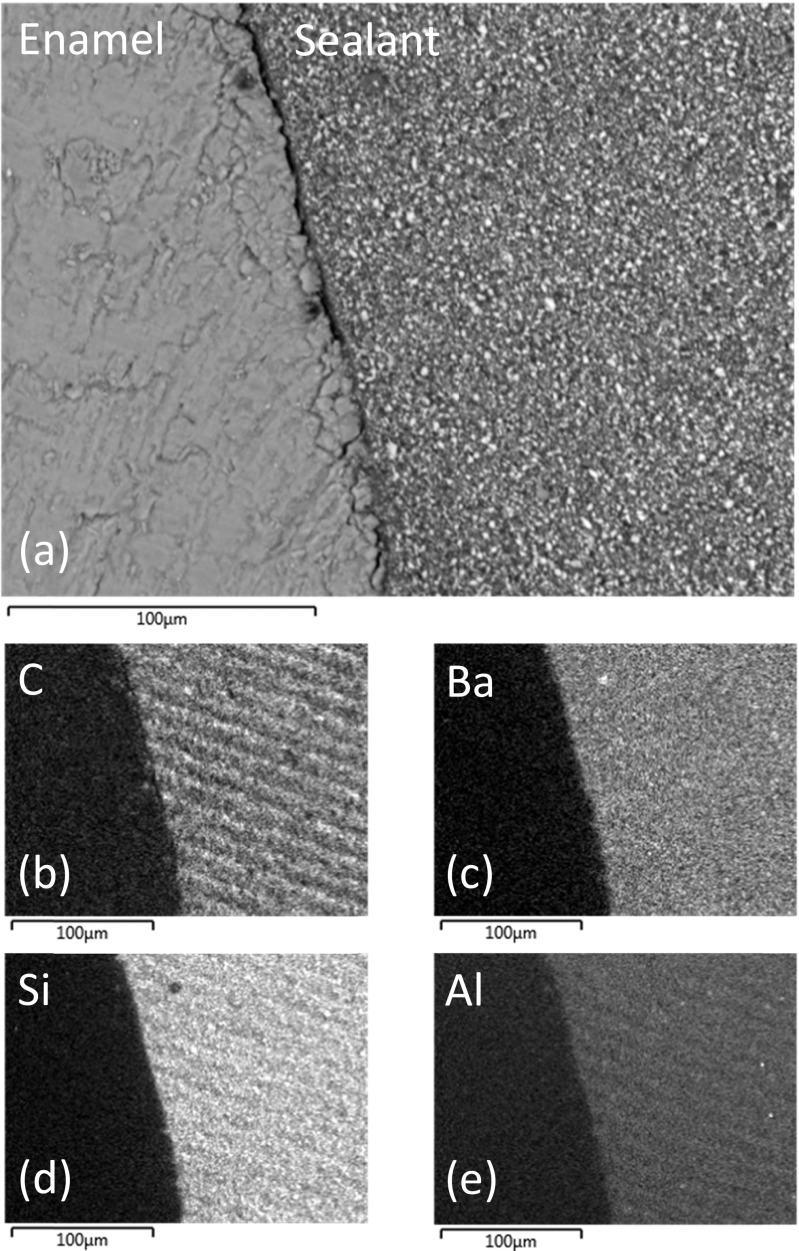
**a** Back-scattered SEM image of UltraSeal XT® hydro™ in contact with acid-etched enamel and corresponding EDX maps of **b** carbon, **c** barium, **d** silicon and **e** aluminium

**Fig. 3:**
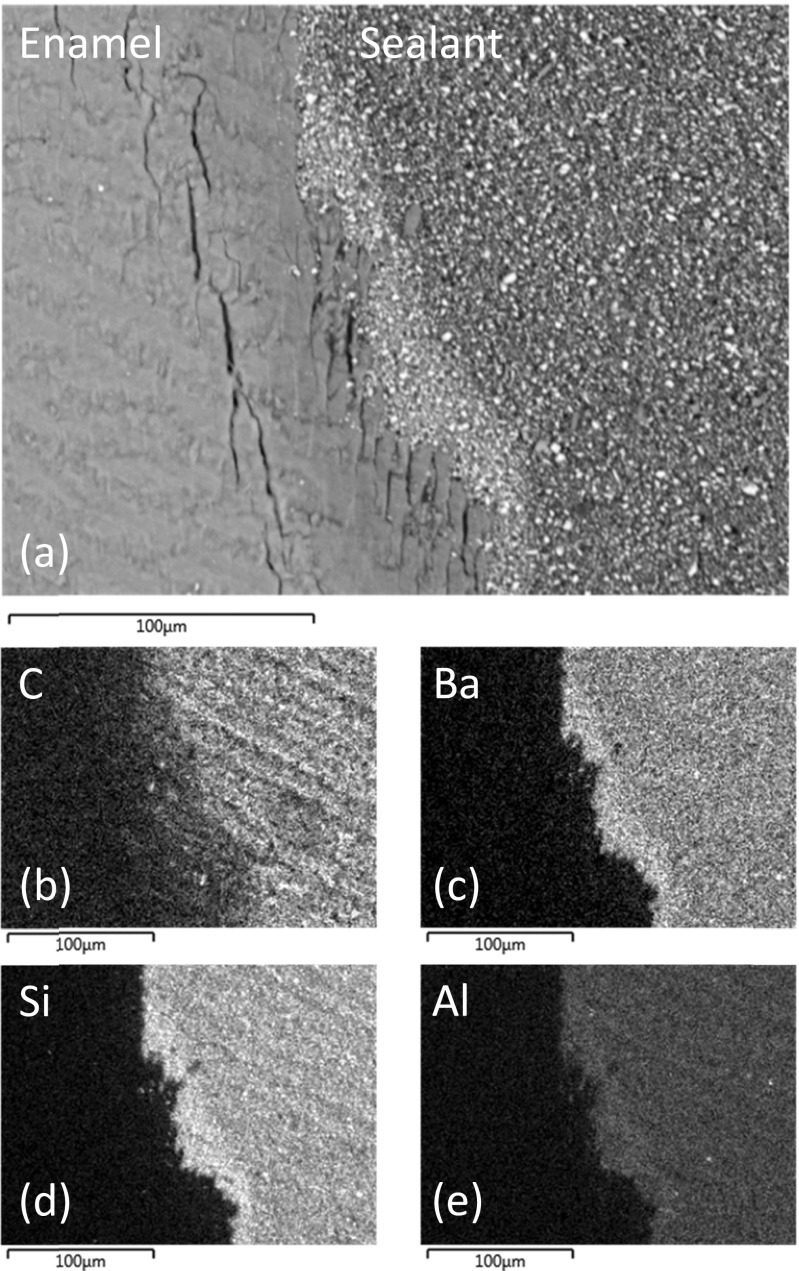
**a** Back-scattered SEM image of UltraSeal XT® hydro™ in contact with lased enamel and corresponding EDX maps of **b** carbon, **c** barium, **d** silicon and **e** aluminium

**Fig. 4:**
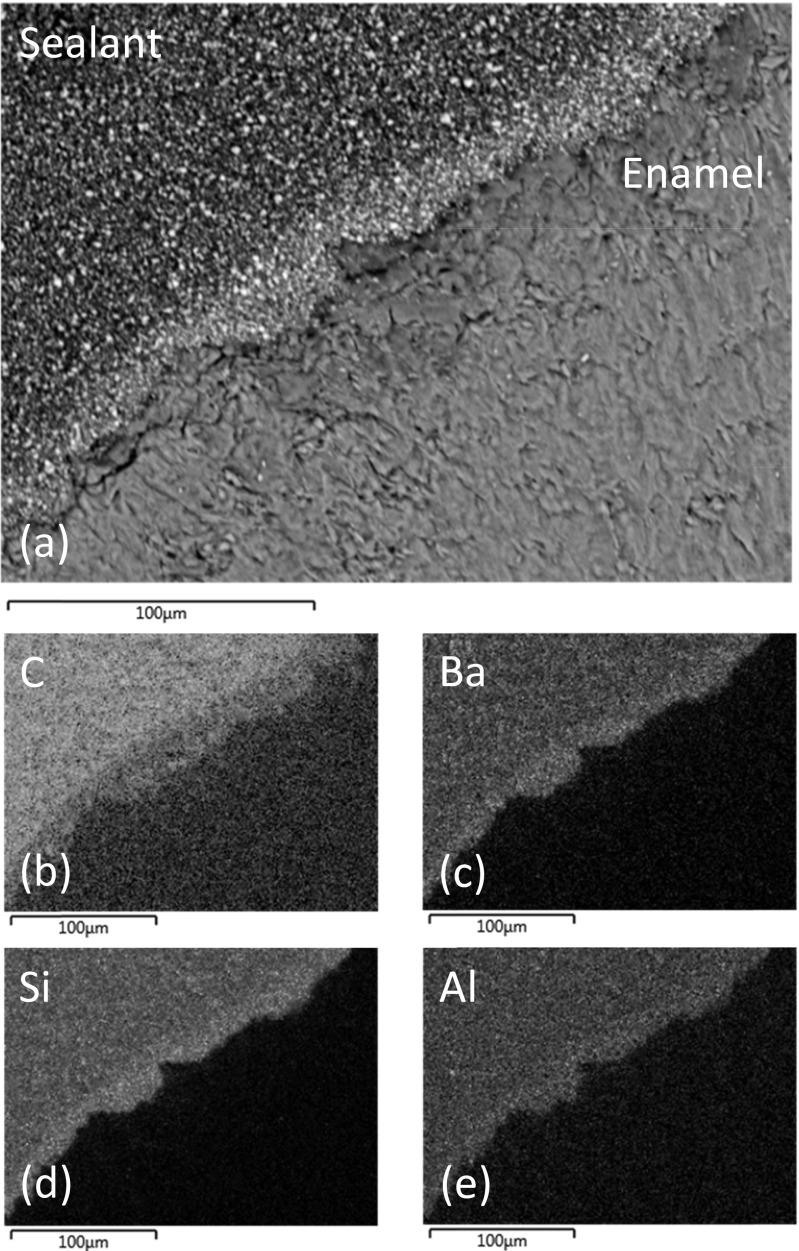
**a** Back-scattered SEM image of UltraSeal XT® hydro™ in contact with sequentially lased and acid-etched enamel and corresponding EDX maps of **b** carbon, **c** barium, **d** silicon and **e** aluminium

Representative back-scattered SEM images of sealed and sectioned teeth from groups I, II and III are shown in Figs. 2a, 3a and 4a, respectively. The lased teeth in groups II and III (Figs. 3a and 4a) presented enhanced surface roughness on a 50–100-μm scale, and these samples also exhibited regions of sub-surface cracking at depths of between 10 and 75 μm from the enamel surface. Figure 2a demonstrates that the particulate inorganic filler within the sealant remained uniformly distributed when the material was placed in contact with the acid-etched enamel surface. Conversely, Figs. 3a and 4a indicate that the enhanced surface roughness of the lased teeth caused a concentrated zoning of the filler particles at the enamel-material interface.

Energy-dispersive X-ray analysis indicated that the major elemental constituents of UltraSeal XT® hydro™ are carbon, oxygen, barium, silicon, aluminium, sodium and phosphorus. EDX maps of carbon, barium, silicon and aluminium, which correspond to the SEM images of the sealed teeth in groups I, II and III, are shown in Figs. 2b–e, 3b–e and 4b–e, respectively. These data confirm that the filler incorporated into UltraSeal XT® hydro™ is principally composed of inorganic barium-, silicon- and aluminium-bearing phases which are preferentially collected at the sealant-enamel interface in group II and III samples.

## Discussion

The principal aim of this study was to evaluate the suitability of different enamel conditioning regimes with respect to the microleakage of a new hydrophilic fissure sealant, UltraSeal XT® hydro™, placed on the occlusal surfaces of molar teeth. To our knowledge, there are currently no other reports on the impact of laser conditioning on the sealing performance of this material. The results have indicated that, under the selected experimental parameters, there is no significant difference in the degree of microleakage between sealed teeth that were traditionally acid etched and those that were subjected to Er:YAG laser ablation (Table 3). This study has also demonstrated that sequential enamel conditioning by laser ablation and acid etching substantially improves resistance to microleakage in comparison with either technique applied separately.

In vitro microleakage tests provide a semi-quantitative evaluation of a dental sealant’s capacity to maintain good marginal adaptation in order to prevent bacterial ingress and caries formation. Although widely applied, the operating parameters of these microleakage tests (e.g. temperature range, dwell time, number of cycles, type of dye, immersion medium) are not standardised [3–9], and caution is required when comparing microleakage results from different studies. These tests typically involve an examination of the depth of penetration of either silver nitrate, methylene blue, rhodamine or fuchsin dye along the sealant-enamel margin after the sealed tooth has been subjected to between 400 and 4000 thermal cycles from 5 to 55 °C with a dwell time of 15, 30 or 60 s. This microleakage study employed ‘average’ thermocycling parameters (2500 cycles between 5 and 55 °C with a dwell time of 30 s) and subsequent immersion in 0.5 % basic fuchsin solution which is considered the most effective dye for revealing microleakage [8, 9].

Recent research presents conflicting reports on the comparative efficacy of laser ablation and acid etching on the retention and microleakage of resin-based fissure sealants [3, 4, 6–9]. In general, current opinion indicates that the more viscous sealants may fail to adequately flow over and adapt to the rougher and more irregular surfaces presented by lased enamel. Other factors that affect the performance of laser ablation in this application are the lack of consensus on the optimal laser operating parameters (e.g. power, pulse rates, working distance, tip diameter) and the potential for laser treatment to cause irregular etching, enamel vitrification and microcracking [9]. The specific pit and fissure morphology, remnant pellicle, organic debris and presence of aprismatic enamel, are also reported to impact upon the relative effectiveness of laser conditioning and acid etching [4, 7].

The results of the present study confirm those of Moshonov et al. [9] who found no significant difference in microleakage between acid etching or Er:YAG laser ablation at higher energy (800 mJ) than those used in this study. Conversely, the findings of the current study conflict with those of Borsatto et al. [13] who report that Er:YAG laser treatment at 120 mJ and 4 Hz afforded poorer marginal sealing than conventional acid etching. In addition, other studies have also indicated that Er:YAG laser conditioning is less effective than conventional acid etching [14, 15]. These observed discrepancies are likely to have arisen from differences in the physicochemical and rheological properties of the various fissure sealants used.

The potential benefit of laser ablation in combination with acid etching to precondition enamel prior to the application of resin-based fissure sealants is also a matter of dispute. Ciucchi et al. [7], Borsatto et al. [13], Youssef et al. [15] and Manhart et al. [16] found that there was no significant difference in hydrophobic sealant microleakage between teeth that were exclusively acid etched and those that were sequentially Er:YAG laser ablated and acid etched. Conversely, Khogli et al. [6] report that a combination of laser ablation and acid etching significantly reduced the extent of marginal leakage for both hydrophobic and hydrophilic sealants compared with conventional acid etching only. Very few studies have been carried out to investigate the impact of laser ablation on the microleakage of flowable hydrophilic sealants, and in this respect, the present study indicates that the combination of laser irradiation and acid etching may afford an advantage over conventional acid etching.

SEM analysis of the teeth in groups II and III of this study which were subjected to Er:YAG laser ablation has revealed enhanced surface roughness and regions of sub-surface microcracking at depths of between 10 and 75 μm. Enhanced surface roughness (up to 155 μm) has been widely reported for lased enamel surfaces [3, 4]. As previously mentioned, the potential benefits of the different scales of surface roughness afforded by various enamel conditioning techniques are reported to depend upon the nature of the selected sealant. In general, it is suggested that more flowable sealants are better suited to the rougher enamel surfaces presented by lased teeth. Incidences of enamel microcracking have also been reported for teeth subjected to laser irradiation [17]; although, it is presently unclear whether these may have a detrimental impact of the longevity of the sealant.

Electron microscopy with energy-dispersive X-ray analysis has revealed that the mineral filler component of UltraSeal XT® hydro™ essentially comprises micrometer-sized particles of inorganic silicon-, aluminium- and barium-bearing phases. This finding conflicts with the information contained in the UltraSeal XT® hydro™ safety data sheet which indicates that this sealant contains aluminium and titanium oxides and does not list silicon- and barium-bearing phases (Table 1) [12]. SEM and EDX analyses have also shown that the increased roughness of the enamel surface of the lased teeth causes a preferential zoning of the filler particles at the enamel-sealant interface.

Potential technical and clinical advantages of laser ablation to prepare occlusal surfaces for the application of fissure sealants include reduced chair time, more effective removal of debris, superior conditioning of narrow fissure zones and aprismatic hydroxyapatite and enhanced acid resistance of lased enamel [3, 4, 7, 10]. A current disadvantage of this technique is the lack of consensus on the optimal operating parameters of the laser to enable controlled regular etching and to minimise enamel vitrification and microcracking [7, 9]. An additional limitation to the possible effectiveness and widespread clinical acceptance of laser conditioning is the lack of fissure sealant materials that are specifically designed to adapt to lased enamel. Current commercially available fissure sealants have been formulated to adhere to acid-etched enamel, and it is now suggested that a new generation of materials is required in order to fully exploit the clinical potential of laser conditioning. In this respect, the combination of laser conditioning and a suitable hydrophilic fissure sealant would afford a significant advantage in paediatric dentistry where chair-time, patient compliance and exclusion of moisture present particular problems for the clinician.

## Conclusions

UltraSeal XT® hydro™ is a new hydrophilic, light-cured, methacrylate-based pit and fissure sealant which has been developed by Ultradent Products, USA. In this study, extracted human molars were conditioned *via* phosphoric acid etching (*n* = 10), laser irradiation (*n* = 10) or sequential laser irradiation and acid etching (*n* = 10) prior to the placement of UltraSeal XT® hydro™. The sealed teeth were then subjected to 2500 thermocycles between 5 and 55 °C and assessed for microleakage *via* fuchsin dye penetration. The Mann–Whitney *U* test indicated no significant differences in microleakage between the individually acid-etched and lased groups (*p* > 0.05); however, teeth treated with a combination of laser irradiation and acid etching demonstrated significantly lower microleakage scores (*p* < 0.001).

Electron microscopy with energy-dispersive X-ray analysis revealed that the mineral filler component of UltraSeal XT® hydro™ essentially comprises micrometre-sized particles of inorganic silicon-, aluminium- and barium-bearing phases. Laser etching was found to increase the roughness of the enamel surface which caused a concentrated zoning of the filler particles at the enamel-sealant interface.

This study has demonstrated that laser ablation may improve the retention of hydrophilic fissure sealants; although, further research is required to optimise lasing parameters and it is suggested that new sealants are needed which are specifically designed to adapt to lased enamel.
